# Primary Subcutaneous Hydatid Cyst of the Abdominal Wall in a Child: A Case Report and Literature Review

**DOI:** 10.7759/cureus.99953

**Published:** 2025-12-23

**Authors:** Hafid Talha

**Affiliations:** 1 Department of Pediatric Surgery, Moulay Ali Cherif Regional Hospital Center, Errachidia, MAR; 2 Laboratory of Health Sciences of Errachidia, Faculty of Medicine and Pharmacy Errachidia, Moulay Ismail University of Meknes, Errachidia, MAR

**Keywords:** abdominal wall hydatid cyst, cystic hydatidosis, echinococcosis granulosis, hydatid cyst, subcutaneous hydatid cyst

## Abstract

Hydatid disease is a parasitic infection that can affect almost any organ, with a clear predominance for the liver and lungs, while primary involvement of subcutaneous tissue is exceptionally rare. We report the case of an eight-year-old boy from an endemic region who presented with a slowly growing, painless mass in the right iliac fossa, without systemic symptoms. Ultrasound revealed a well-defined, purely cystic lesion consistent with a Gharbi type I hydatid cyst. Thoracoabdominal CT scan showed no additional hydatid localizations, particularly in the liver or lungs. The lesion was completely excised through a right iliac fossa approach without intraoperative complications, and albendazole therapy was administered. A review of the literature identified only a small number of abdominal wall hydatid cysts located in the subcutaneous tissue, with very few paediatric cases and most associated with concomitant visceral disease. This case illustrates that hydatid disease should be considered in the differential diagnosis of any subcutaneous cystic mass in patients from endemic areas, even in the absence of hepatic or pulmonary involvement.

## Introduction

Hydatid disease is a parasitic infection that can involve almost any organ of the human body, although the liver and lungs are most frequently affected, while other sites, such as the subcutaneous tissue, are rarely encountered [1]. Pastoral and poor rural areas, where people keep livestock in close proximity to dogs, are the most affected regions [2].

The liver and lungs act as natural mechanical filters for parasitic oncospheres, making it difficult for the parasite to reach atypical locations without primary involvement of these organs [1]. Solitary primary subcutaneous localization is therefore very rare, and its exact incidence remains unknown [3]. The diagnosis of hydatid disease relies on imaging and serological investigations [4]. Surgery remains the preferred therapeutic modality; however, recurrences are frequent following resection [5].

Herein, we report the case of an eight-year-old boy with hydatid disease of the anterior abdominal wall, confined to the subcutaneous tissue and without primary hepatic or pulmonary involvement, who was successfully treated with complete surgical excision.

## Case presentation

**Figure 1:**
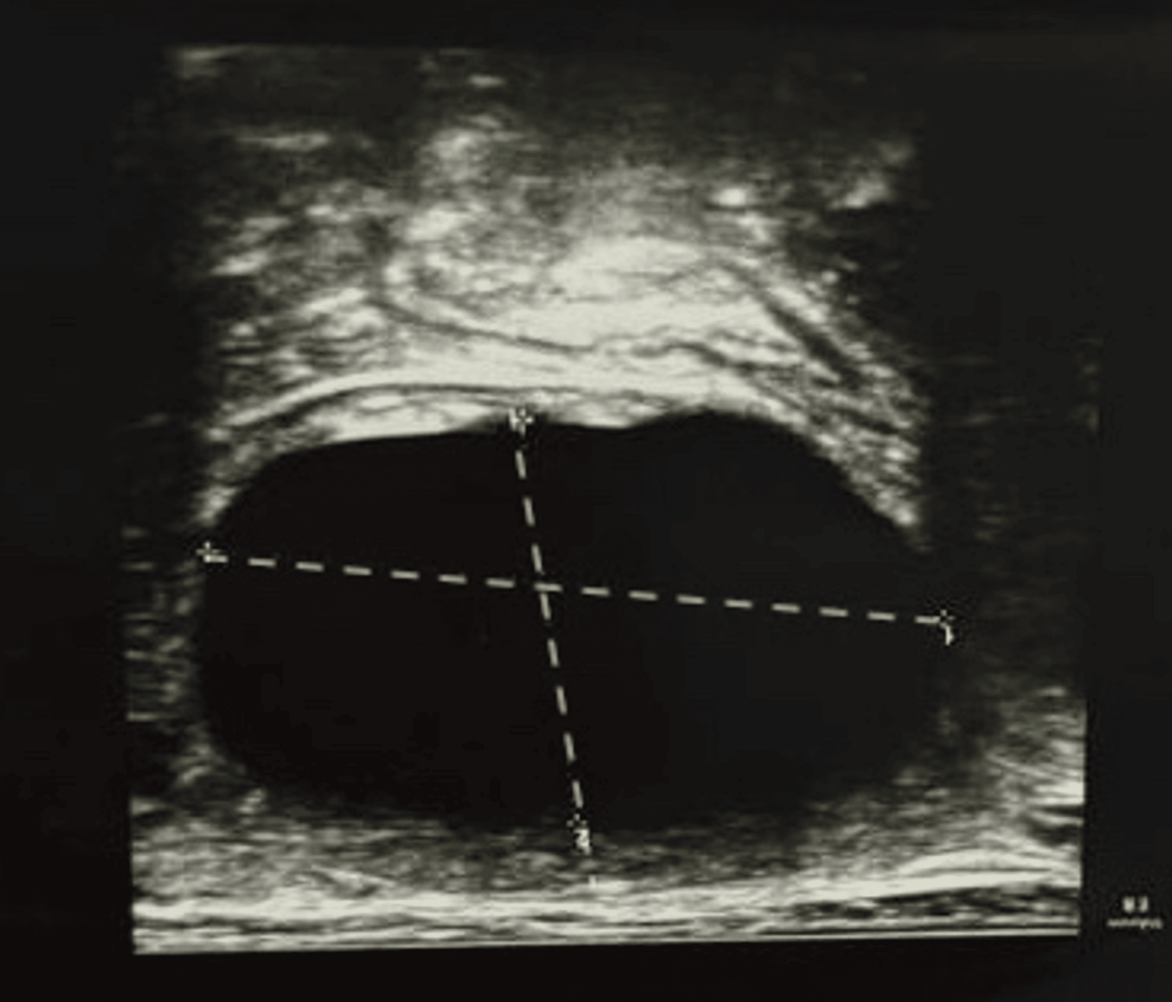
Ultrasound findings of the abdominal lesion. Ultrasound of the abdominal lesion showing a purely cystic lesion with no solid components or membranous structures, consistent with a stage I hydatid cyst of Gharbi classification.

An eight-year-old boy presented with a slowly growing, painless mass in his right iliac fossa, without associated fever. The parents reported a history of contact with dogs. Initial ultrasound examination showed a fluid-filled cystic lesion without solid components, measuring 5x4.5x4cm consistent with a stage I cyst according to the Gharbi classification [6] (Figure 1). A complete imaging workup with a thoracoabdominal CT scan did not reveal any additional hydatid localizations, particularly in the lungs or liver. Admission laboratory results were within normal range and are summarized in Table 1.

**Table 1:** Summary of laboratory results at admission. Admission laboratory investigations were all within the normal range, with no signs of inflammatory reaction.

Parameters	Patient’s results	Reference values
Hemoglobin (g/dL)	13.2	13–17
White blood cells (×10⁹/L)	7.4	4.0–10.0
Neutrophils (%)	62	40–75
Platelets (×10⁹/L)	260	150–400
C-reactive protein (mg/L)	6	< 5
Creatinine (mg/dL)	0.9	0.6–1.2
Urea (mg/dL)	24	15–45
Sodium (mmol/L)	139	135–145
Potassium (mmol/L)	4.2	3.5–5.0
Aspartate aminotransferase (U/L)	22	< 35
Alanine aminotransferase (U/L)	25	< 45
Glucose (g/dL)	0.9	0.7–1
Prothrombin activity (%)	95	70-100

**Figure 2:**
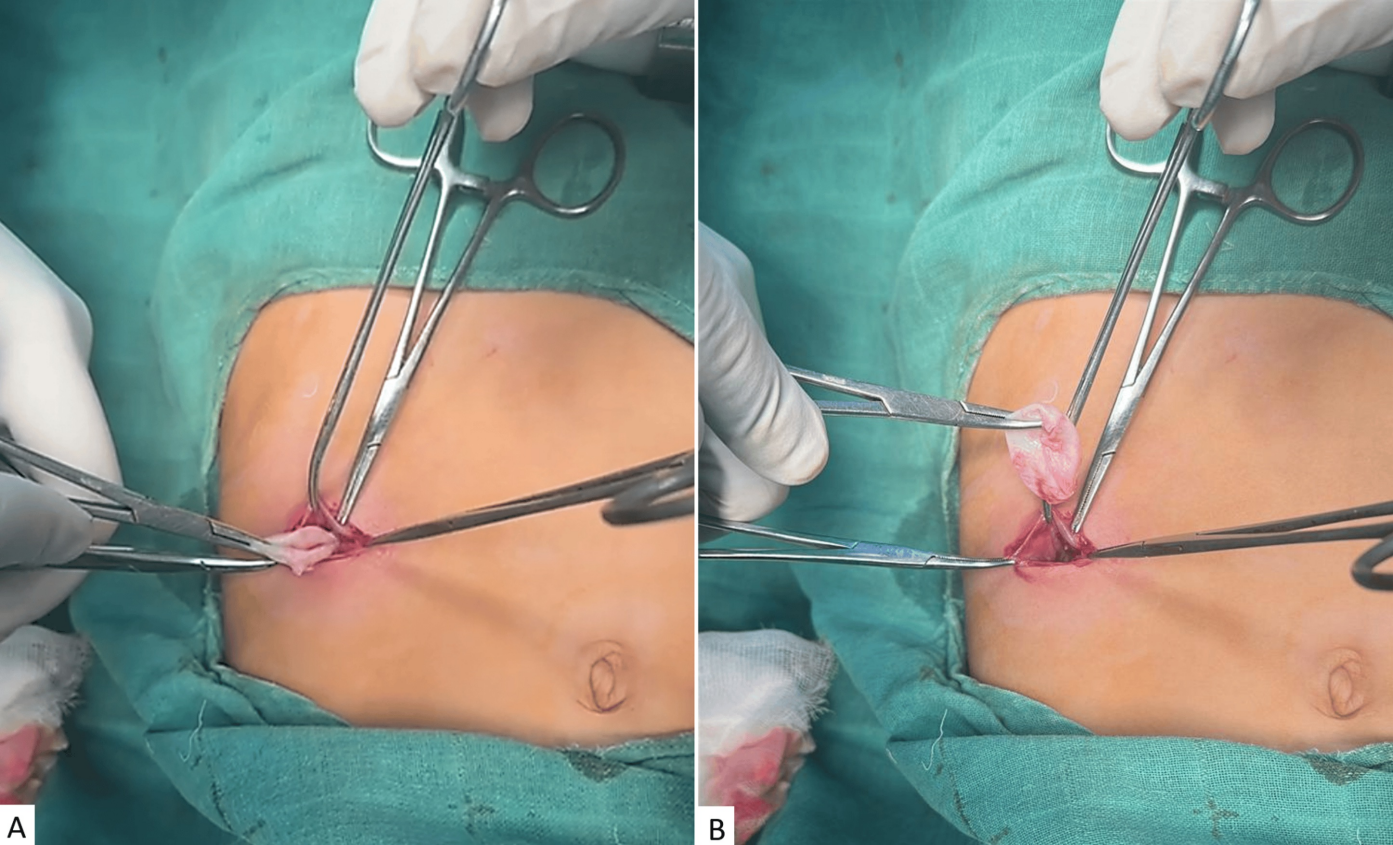
Intraoperative photographs showing removal of the cyst through a right iliac fossa incision (A and B).

A hydatid cyst was strongly suspected, and the lesion was surgically excised without intraoperative complications. The operative field was first isolated with pads soaked in a scolicidal agent, followed by cyst puncture and controlled aspiration using a trocar aspirator to minimize spillage. After evacuation, the puncture orifice was enlarged to allow exposure of the cyst cavity, and the hydatid membrane was gently grasped and extracted without rupture (Figure 2).

No postoperative complications were detected. The patient received albendazole therapy; preoperative albendazole was administered as adjuvant therapy (10-15 mg/kg/day in two doses) prior to surgery to reduce cyst viability and the risk of recurrence/spillage. At the one-year follow-up, the patient remained asymptomatic, with no clinical or radiological signs of recurrence.

## Discussion

Hydatidosis is a zoonosis caused by *Echinococcus granulosus*, a cestode belonging to the family Taeniidae [7]. Hydatid disease is considered a major public health problem, particularly in developing countries [8]. It remains endemic in regions where livestock raising is common, including Mediterranean countries, the Middle East, India, Oceania, and South America [9]. Humans act as accidental intermediate hosts, with transmission occurring via orofecal contamination. After intestinal absorption, *Echinococcus* larvae cross the intestinal wall and reach the liver and other organs through the portal circulation [2].

The liver and lungs are the most frequently affected sites of hydatid cysts. Other locations, including the kidneys, spleen, bones, and brain, are less commonly affected [8]. Although hydatid disease can theoretically involve any organ, primary hydatid cysts arising in soft tissues such as the subcutaneous tissue, without concomitant hepatic or pulmonary involvement, are exceedingly uncommon [2]. The reported incidence of subcutaneous hydatid cysts is approximately 2% of all hydatid cysts, and most of these are associated with liver and/or lung disease; primary subcutaneous hydatid cysts are therefore exceedingly rare [9]. Patmano et al. reported only one abdominal wall localization among eight subcutaneous hydatid cysts in their series [10].

In our review of the literature, we identified several dozen subcutaneous localizations, including both isolated and disseminated forms. However, to the best of our knowledge, only five cases have described a similar localization to ours, in the subcutaneous tissue of the abdominal wall; among these, just one involved a pediatric patient (Table 2). Notably, that pediatric case was associated with primary pulmonary disease, in contrast to our patient, who presented with an isolated subcutaneous abdominal wall hydatid cyst.

**Table 2:** Comparative summary of reported cases of hydatid cysts in the subcutaneous tissue of the abdomen, with or without associated lung and/or liver involvement.

Case Report	Age/sex	Location	Dissemination	Primary/Other disease
Ousadden et al. [4]	70 F	Para-umbilical abdominal wall	Isolated	Complete surgical removal of the cyst
Gulmez et al. [9]	60 F	Left periumbilical region of the abdominal wall	Isolated	Complete surgical removal of the cyst
Patmano et al. [10]	45 M	Right upper quadrant of abdominal wall	Isolated	Complete surgical removal of the cyst
El Haissoufi et al. [11]	6 M	Right posterolateral abdominal wall	Associated to lung hydatid disease	Surgical excision of the cyst + albendazole therapy
Bedioui et al. [12]	70 F	Hypogastric region	Isolated	Complete surgical removal of the cyst
Present case	8 M	Right iliac fossa	Isolated	Complete surgical excision of the cyst + albendazole therapy

Passage of the parasite from the portal vein into the systemic circulation is difficult because of the well-developed capillary networks of the liver and lungs, which act as an effective filter [2]. The exact mechanisms underlying primary hydatid cysts, outside the liver and lungs, at rare sites, including the abdominal wall, remain unclear. The most widely accepted hypothesis is subcutaneous colonization by ingested eggs after they have traversed the hepatic and pulmonary filters [8]. A small number of ova may pass through the liver and reach the lungs and systemic circulation, thereby causing hydatid disease in other organs [4]. Alternatively, parasites may bypass the portal-liver filter by using lymphatic pathways or venous shunts to gain direct access to the systemic circulation [8]. Direct extension from adjacent organs represents another possible mechanism for involvement of unusual sites [4].

The most common clinical manifestation is a painless, slowly growing mass without inflammatory signs [13], with a wide range of nonspecific symptoms that are largely related to the localization and size of the cyst [9]. The diagnosis of hydatid cyst is based mainly on imaging modalities, including ultrasonography, CT scan, and magnetic resonance imaging (MRI), together with serological tests (e.g., indirect hemagglutination and enzyme-linked immunosorbent assay (ELISA)), which may be used; however, negative serology does not exclude hydatid disease. Therefore, serological testing was not performed in our case.

In some cases, the diagnosis is only confirmed by preoperative biopsy or on postoperative histopathological examination [8]. MRI provides a detailed assessment of soft tissue structures and the degree of extension [2]. Imaging also helps determine the stage of the hydatid cyst and to exclude differential diagnoses. In subcutaneous locations, the differential diagnosis includes other soft tissue masses, mainly abscesses, dermal and epidermal cysts, and lipomas [9]. Preoperative recognition of hydatid cysts is crucial, as the condition must be distinguished from other entities with similar presentations [2]. Diagnosis may be particularly challenging when classical sites such as the liver and lungs are not involved and when epidemiological risk factors are absent.

Complete surgical excision of the cyst is the treatment of choice in the majority of cases [8], considering the exact site of the lesion, the proximity of critical structures, and any systemic involvement [2]. Surgical management carries specific risks, primarily related to the operative procedure itself and to potential cyst rupture, which may result in anaphylaxis, dissemination, and local recurrence [8]. When the complete resection of the cyst is not possible, all the cyst content should be carefully evacuated [9].

Percutaneous approaches have also been reported as effective treatment options for hydatid cysts of the lungs, kidneys, orbit, and parotid glands [8]. Other management strategies include aspiration followed by injection of a scolicidal agent and re-aspiration, systemic albendazole therapy, and surveillance in selected cases [2]. However, benzimidazoles appear to be less effective in muscle and renal hydatid cysts because of their low concentrations within cyst fluid [1].

In our case, the cyst was managed surgically, with no perioperative complications and no evidence of recurrence at the one-year follow-up.

## Conclusions

Hydatid disease remains a common parasitic infection that should be considered in the differential diagnosis of any subcutaneous mass, particularly in patients from endemic regions. Radiological assessment of the liver and lungs is essential to detect disseminated disease. The treatment of choice is complete surgical excision of the cyst to minimize the risk of recurrence. Through this case, we aim to raise awareness of hydatid disease and highlight that unusual subcutaneous localizations may occur even in the complete absence of hepatic or pulmonary involvement.
